# High frequency emergency department use and heterogeneity of reasons for attendance by children and young people: a retrospective cohort study

**DOI:** 10.1136/bmjpo-2025-003907

**Published:** 2026-02-26

**Authors:** Akshay Kumar, Rebecca M Simpson, Kerryn Husk, Graham D Johnson, Chris Burton

**Affiliations:** 1 University of Leeds, Leeds, UK; 2 ScHARR, The University of Sheffield, Sheffield, UK; 3 NIHR CLAHRC South West Peninsula (PenCLAHRC), University of Plymouth, Plymouth, UK; 4 Emergency Department, University Hospitals of Derby and Burton NHS Foundation Trust, Derby, UK; 5 School of Medicine, University of Nottingham, Nottingham, UK

**Keywords:** Statistics, Children

## Abstract

**Objective:**

To quantify patterns of emergency department (ED) use over two consecutive 12-month periods among children aged 15 and under, and to assess heterogeneity of reasons for attendance in high-frequency users.

**Design:**

Population-based retrospective cohort study of routinely collected ED data.

**Setting:**

EDs in the Yorkshire and Humber region, UK, from 31 March 2014 to 1 April 2017.

**Patients:**

Children aged 15 and under with ≥1 ED attendance.

**Main outcome measures:**

Proportion with ≥7 attendances over 2 years and heterogeneity of diagnostic reasons quantified by the Herfindahl index.

**Results:**

The cohort included 71 143 individuals. Although only 13.6% were high-frequency attenders in the first year, over half (55.1%) of these made at least one attendance in the second year. A subset (14.1%) remained high-frequency attenders across both years and were more likely to belong to the most deprived deprivation category. Children aged 8–12 were more likely to attend for injury-related issues and showed lower heterogeneity in reasons for attendance, while infants under age 1 had more illness-related attendances and greater heterogeneity.

**Conclusions:**

A notable proportion of children and young people frequently attend EDs over a 2-year period. This study introduces a method for quantifying heterogeneity in reasons for attendance, which may support future predictive modelling using electronic health records to identify and support high-frequency ED users.

**WHAT IS ALREADY KNOWN ON THIS TOPIC**

- Some children frequently use the emergency department, but the persistence of this high use over consecutive years and the diversity of reasons for their visits were poorly understood.

**WHAT THIS STUDY ADDS**

- This study shows that a notable proportion (14.1%) of high-frequency users remain so over 2 years, are often from deprived areas, and introduces a method showing infants have more varied reasons for attendance than older children.

**HOW THIS STUDY MIGHT AFFECT RESEARCH, PRACTICE OR POLICY**

- This new method for quantifying visit heterogeneity can improve predictive modelling to identify at-risk children and help target specific clinical and public health interventions.

## Background

Urgent and emergency care (UEC) services such as emergency departments (EDs) have experienced huge pressure in recent years from rising demand for care.^1^,^4^ Particular attention has been given to the use of UEC services by children and young people (C&YP), especially in light of recent surges in emergency care activity within this group—such as the notable increase in ED attendances related to scarlet fever and invasive group A streptococcus in 2022/2023.^5^ One study exploring healthcare use by C&YP found that while GP consultations generally decreased for C&YP between the years 2007/2008 and 2016/2017, ED attendance rates increased across this demographic over the same period.^6^ Understanding how and why C&YP access UEC services is essential, as their healthcare needs often differ significantly from those of adults. This is particularly true for younger children, who are more vulnerable and reliant on parents or caregivers and often require specialist support.^7^ When considering high frequency ED use by C&YP, most studies report attendance data from a single year. However, analysing attendance data over multiple years can provide useful insights into ED attendance patterns for those defined as high frequency users over a number of years.^8 9^

C&YP attend EDs for a wide variety of reasons, ranging from injury to illness-related attendances.^10^ For frequently attending C&YP, although there is evidence to suggest these individuals were more likely to be living with one or more long-term conditions,^11^,^14^ little is known about the heterogeneity of reasons for attending EDs. Exploring the heterogeneity of reasons for attending the ED is important to understand attendance behaviours in individuals and may be utilised as a predictor of future frequent ED use.

The aims of this study are to:

1. Measure ED attendance patterns among C&YP over a 2-year period.
2. Identify common reasons for ED attendances among high-frequency attenders.
3. Examine the heterogeneity of reasons for ED use among high-frequency attenders.

The authors would like to acknowledge that parts of the content presented in this manuscript are drawn from Dr Akshay Kumar’s doctoral thesis titled ‘*Examining patterns of urgent and emergency care service use by children and young people’ (PhD thesis, University of Sheffield, 2023*). Specifically, this manuscript is adapted from chapter 9 of the thesis.^15^

## Methods

### Study design

Retrospective cohort study.

### Data used

This study used data collected from the ‘Connected Health Cities: Data linkage of urgent care data’ study (known as the ‘CUREd research database’).^16^ The CUREd Database has approval from the National Health Service (NHS) Research and Ethics Committee, overseen by the NHS Health Research Authority’s Research Ethics Service, and from the NHS Health Research Authority, directly, to receive health and social care data without patient consent for patients of UEC services in Yorkshire and Humber. The CUREd research database holds data from NHS 111 calls, emergency ambulance incidents, ED attendances and emergency admissions to hospitals in the Yorkshire and Humber region of the UK and contains an anonymised patient identifier code to facilitate linkages across the datasets. This study extracted data from the ED dataset which contained patient records between 31 March 2014 and 01 April 2017 (consisting of items mandated by the national Commissioning Dataset-CDS) for attendances made to the 13 participating Hospital Trusts’ EDs. An ED attendance was defined as an unplanned ED attendance made to a type 1 ED, type 2 ED or other type of ED with designated accommodation for the reception of ED patients.^17^ Attendances made to services that were mainly/entirely appointment based were excluded from this analysis.

### Patient and public involvement

Patients were not directly involved in the planning or execution of this research which analysed routinely collected healthcare data. However, PPI plays a pivotal part in the conceptualisation and collection of the CUREd research database and utilises the assistance of a Data Release Committee (DRC) which acts as an oversight panel for the CUREd platform, including patient and public representation, healthcare stakeholders, and information governance specialists. The DRC reviewed this study which was designed to be a secondary data analysis based on a noted literature gap.

### Data management

#### Data extraction

The data extraction, cleaning, linking and statistical analyses were performed using R V.4.2.1 (R Software Foundation) and packages such as ‘dplyr’ and ‘alluvial’. The following items were extracted from the ED dataset: Anonymised patient identifier, encrypted attendance record identifier, date/time of ED attendance, sex of patient, incident Index of Multiple Deprivation (IMD) quintiles,^18^ age at first attendance, the hospital/ED an attendance was made to and the primary reason for attendance (primary diagnosis).

#### Cohort preparation

Due to low rates of primary diagnosis coding in some EDs, only EDs with ≥70% non-missing primary diagnosis codes were included. This was due to several factors: suboptimal diagnosis code entry by clinicians, changes in diagnosis coding systems at some hospitals during the study period, and limited information provided under the older Hospital Episode Statistics (HES) coding framework. This resulted in four EDs (two university teaching hospitals and two district general hospitals) being retained. The cohort included individuals aged 15 and under who made an ED attendance between 31 March 2014 and 01 April 2015. The date of their first attendance was used to define the start of their 2-year observation period. All subsequent attendances within the next two consecutive 12-month periods were included. High-frequency attenders were defined as individuals with ≥3 attendances in a given 12-month period.^19^

#### Sensitivity analysis

Other cut-off points for missing reason for attendance codes were explored as part of a sensitivity analysis using a threshold of 50% and 90%, respectively. For the sensitivity analysis using a 50% threshold, the proportion of missing values in the data meant some reasons for attendance were not captured. Similarly, reasons for attendance were collated for hospitals with 90% non-missing values. Unfortunately, this produced too few attendances, meaning calculations would display CIs too large to make any statistically justifiable conclusions. Hence, a threshold of 70% was chosen to use in this analysis.

#### Reason for attendance

We extracted the primary reason for attendance, where recorded, for each ED visit. Between 2014 and 2017, three diagnosis coding systems were employed across EDs in England: the legacy HES Accident & Emergency (A&E) 5-character or 6-character codes used for ED-specific reporting; ICD-10 (International Classification of Diseases) codes, primarily applied to admitted patients; and Systematised Nomenclature of Medicine–Clinical Term, introduced with the Emergency Care Data Set in 2017 but not yet routinely adopted during this period. Across the four EDs included in this analysis, two diagnosis coding systems were in use: one site employed ICD-10 codes, while the remaining three used the legacy HES A&E 5-character or 6-character coding system. The coexistence of these systems, combined with inconsistent coding practices, contributed to heterogeneity and missing or non-specific diagnosis entries. For HES-coded data, entries of 1–3 characters were excluded due to ambiguity, as these may have represented anatomical side or region rather than a clinical diagnosis. As the ICD-10 codes provided more detail regarding the reasons for attendance than the 26 HES categories, these codes were grouped into 14 categories based on the ICD-10 (V.2016) disease classifications (online supplemental appendix). With the assistance of medical professionals, we then mapped the HES codes to the ICD-10 codes and provided a new unique single letter code for each of the final categories (see appendix, for the final 14 categories and HES/ICD-10 codes). Attendance codes were then combined into a character string for each patient (eg, an individual making four attendances in their observation period may have the following attendance string ‘AABC’).

### Statistical analysis

#### Exploring ED use over a 2-year period

Individuals were grouped by number of attendances in each 12-month period (year 1: 1, 2, or ≥3; year 2: 0, 1, 2, or ≥3). Alluvial plots were used to visualise transitions in attendance frequency across the 2 years, stratified by age group (<1, 1–4, 5–9, 10–15) and IMD status (more deprived: IMD 1–2; less deprived: IMD 3–5).

#### Exploring reasons for attendance

Attendance reasons were summarised for three groups: (1) individuals with only one attendance, (2) high-frequency attenders in both years and (3) all others. The proportion of injury-related versus illness-related attendances was plotted by age using boxplots.

#### Heterogeneity of reasons for ED attendance

To quantify the heterogeneity of the reasons for attendances in this cohort, a Herfindahl index was computed for each individual.^20^ The Herfindahl index provides a value between 0 and 1 and relates to the number of distinct attendance reasons across their attendance history. For a sequence of attendances including N different categories; and where P*_i_* represents the proportion of attendances belonging to the *i*th category:


$$Herfindahlindex=\sum_{i=1}^{N}P_{i}^{2}$$

A value close to 1 indicates the patient made a large proportion of attendances for the same reason (low heterogeneity) and a value closer to 0 indicates the patient made attendances for different reasons (high heterogeneity). The Herfindahl index was computed for individuals making 7+ attendances within their 2-year observation period, as the number of attendances an individual made was unlikely to confound the Herfindahl index over this threshold. A sensitivity analysis exploring other thresholds was conducted (online supplemental appendix). Box plots were produced to visualise the Herfindahl index across age and IMD status. A linear regression model was computed to test the association between an individual’s Herfindahl index and their characteristics (sex, age and IMD status). For this regression analysis, age was categorised to <1, 1–4, 5–7, 8–12 and 13–15.

## Results

### Patient and attendance characteristics

The study cohort consisted of 71 143 individuals who collectively made 163 814 ED attendances, with a median of 2 attendances per person over their individual 2-year observation periods. Attendances over the study period ranged from a low of 17 686 at the least-visited ED to a high of 52 284 at the most-visited ED. Table 1 shows the cohort age, sex and IMD quintile. Of the 71 143 individuals included in this study, the majority were under the age of 4 (29 996, 42.2%) and belonged to the most deprived IMD category (33 647, 47.3%).

**Table 1:** Patient characteristics (full patient cohort)

Characteristics	Patient Cohort N=71 143	
	n	%
Sex		
Male	39 444	55.4
Female	31 690	44.5
Missing	9	0.1
Age group		
<1	7298	10.3
1–4	22 698	31.9
5–9	17 970	25.2
10–15	23 177	32.6
Missing	0	0
Ethnicity		
White	49 462	69.5
Asian	11 965	16.8
Black	409	0.7
Mixed ethnicity	1344	1.9
Other ethnicities	1602	2.2
Missing	6361	8.9
IMD status		
1 (most deprived)	33 647	47.3
2	12 740	17.9
3	8690	12.2
4	8626	12.1
5 (least deprived)	7003	9.8
Missing	437	0.7

IMD, Index of Multiple Deprivation.

### Exploring ED use over a 2-year period

**Figure 1:**
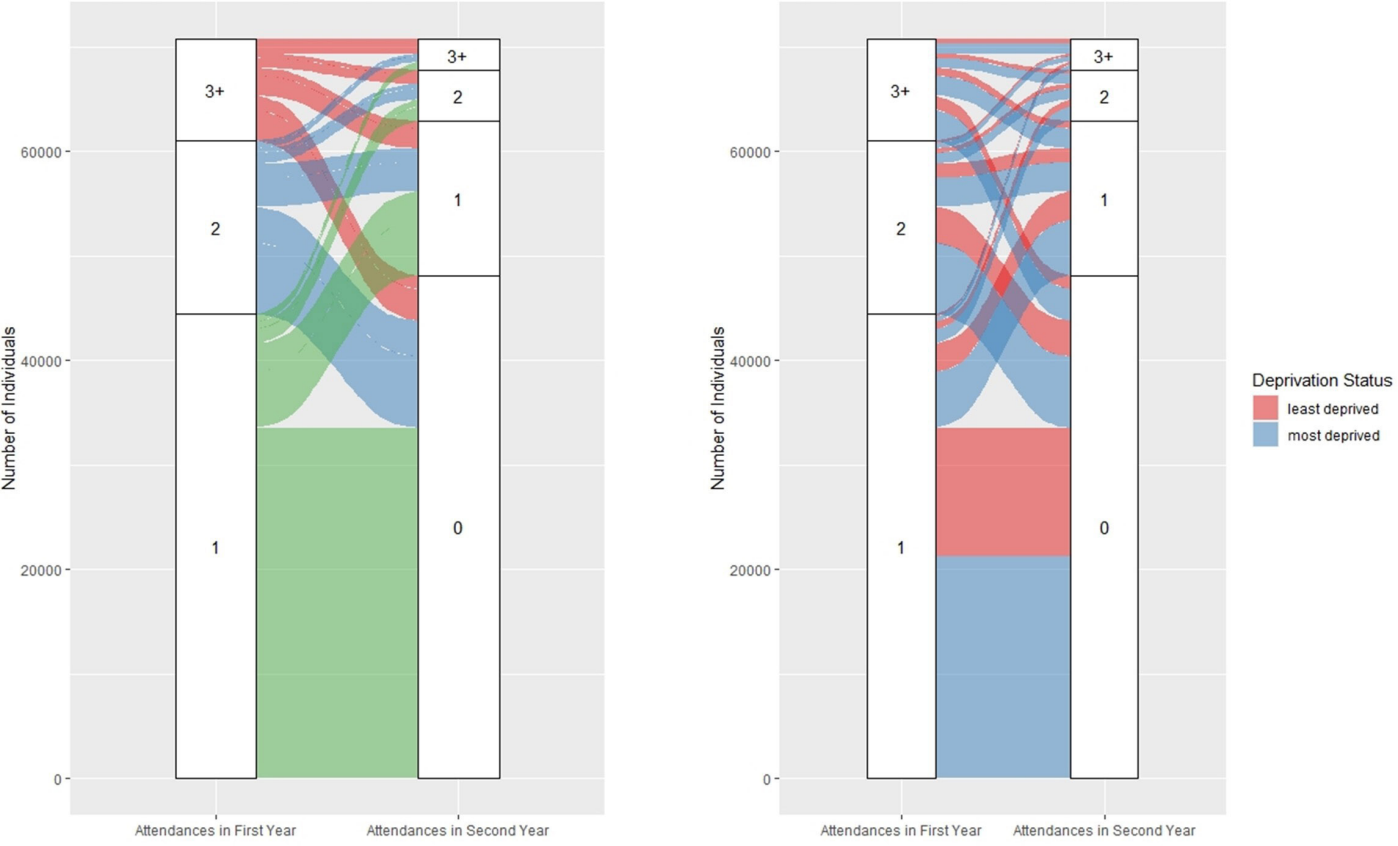
Number of attendances made in each observation year by cohort (deprivation status).

All individuals had an index attendance in the first year. Of these, 33 856 (47.6%) had no further attendances during the 2-year follow-up. A total of 10 936 (15.4%) made one attendance in year 1 and at least one additional attendance in year 2. High-frequency attenders (≥3 attendances in year 1) accounted for 9700 individuals (13.6%), with 5345 (55.1%) of them making at least one attendance in year 2. Notably, 1369 (14.1%) of these individuals were also high-frequency attenders in year 2. Figures1  2 illustrate attendance patterns across the 2 years, stratified by deprivation and age groups. Individuals who were high-frequency attenders in both years were more likely to be from more deprived IMD categories (73.6%; 95% CI 71.2% to 76.0%) compared with those with only one attendance over 2 years (62.7%; 95% CI 62.2% to 63.2%). Among high-frequency attenders in year 1, a notable proportion were infants under age 1 (n=1649; 17%). Of these, 275 (16.7%) remained high-frequency attenders in year 2, compared with 331 (10.6%) of those aged 1–4.

**Figure 2:**
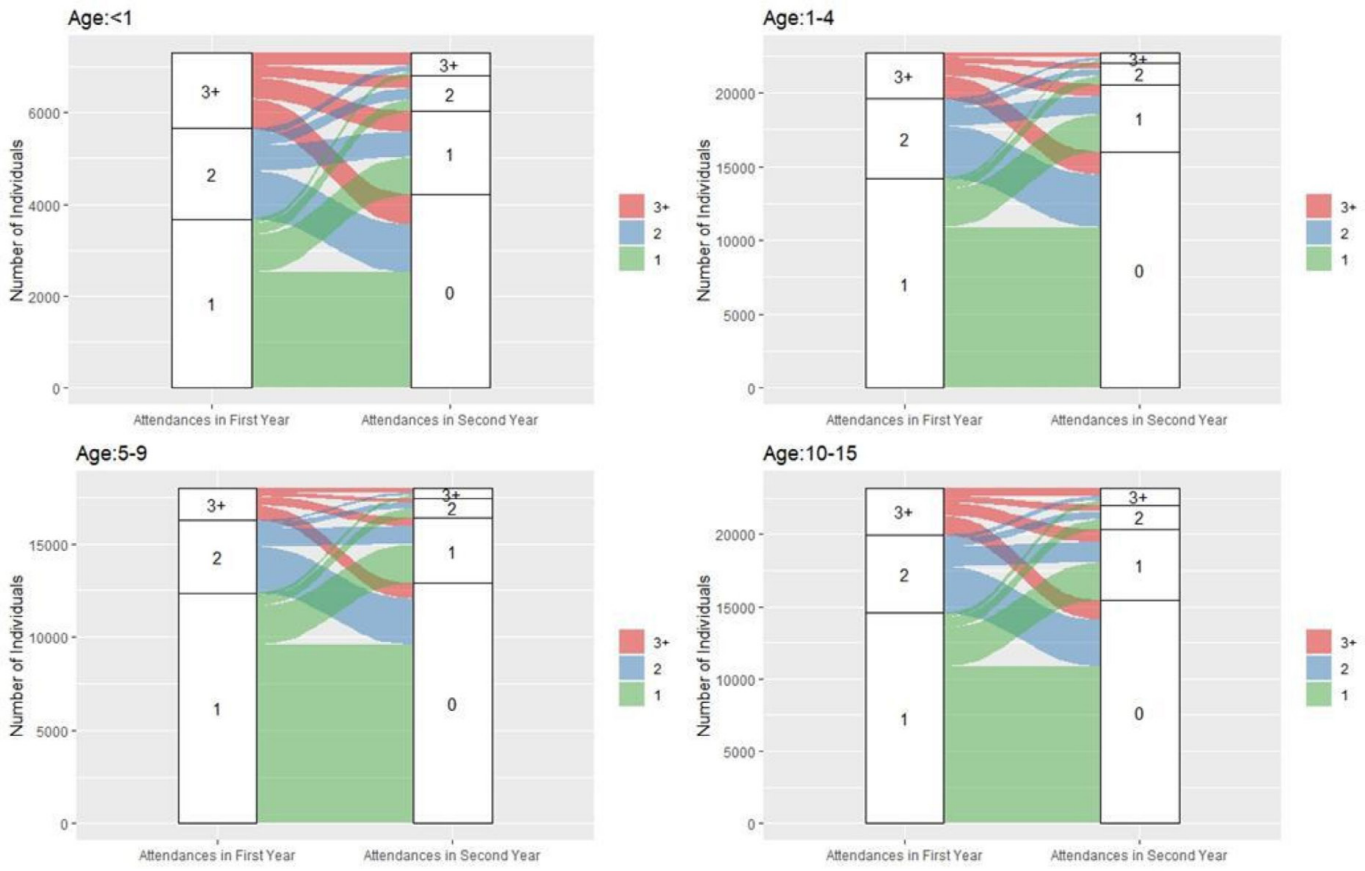
Number of attendances made in each observation year by cohort age (colour corresponds to the number of attendances made in the first observation year).

### Reasons for ED attendance

**Figure 3:**
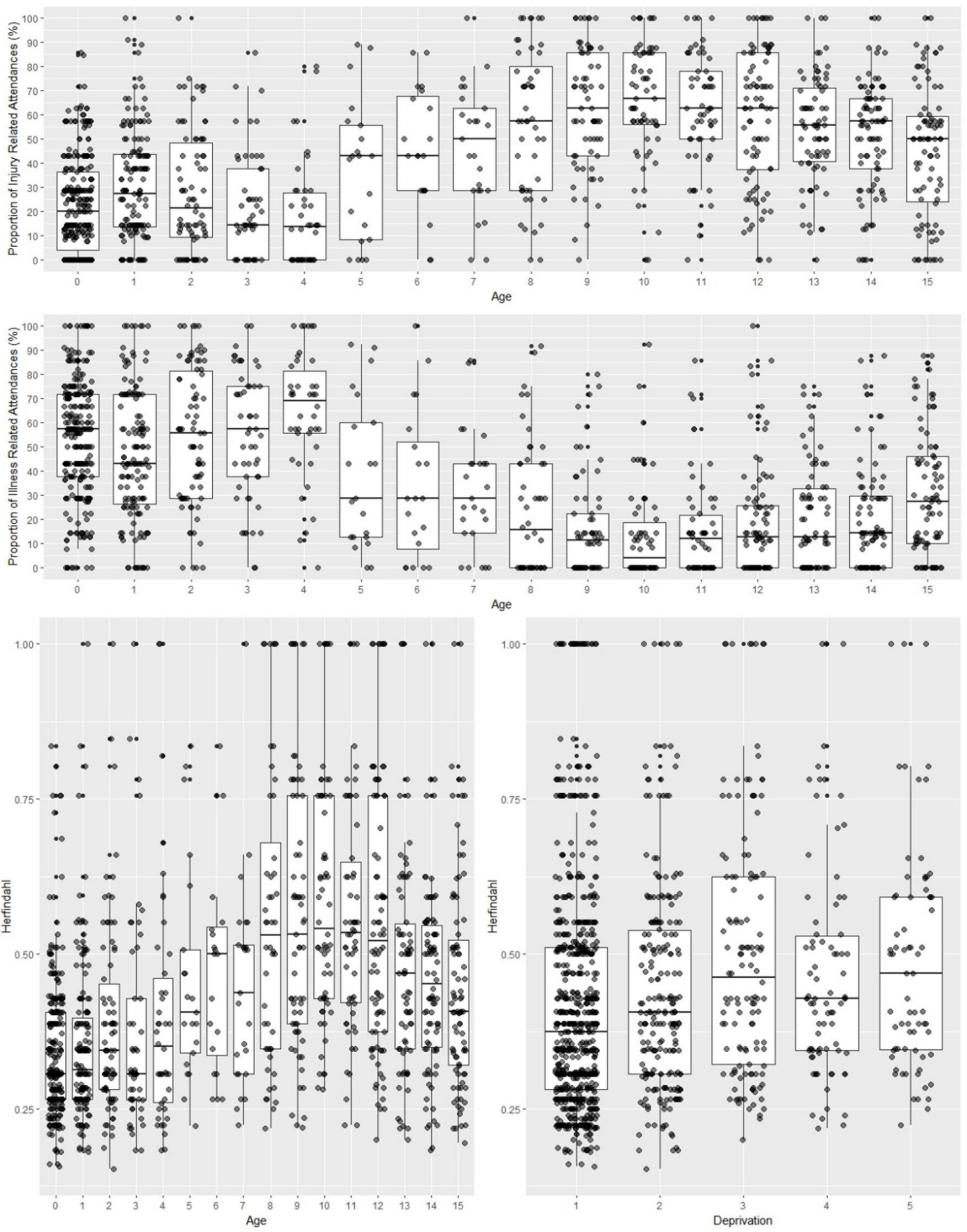
Proportion of injury and illness-related ED attendances by age and heterogeneity (Herfindahl Index) of reasons for attendance by age and deprivation (1=most deprived, 5=least deprived) for high frequency attenders. ED, emergency department.

Reasons for attendance were broadly categorised as injury or illness. Of the total attendances, 72 155 (44%) were injury-related and 38 511 (23.5%) were illness-related (table 2). Figure 3 presents a boxplot of injury and illness-related attendances by age. Younger individuals were more likely to attend for illness-related concerns, while older individuals more frequently attended for injuries. Among high-frequency attenders in both years, injury-related attendances were less common (35%; 95% CI 34.0% to 35.8%) compared with those with a single attendance (47.2%; 95% CI 46.8% to 47.9%). Conversely, illness-related attendances were more prevalent among high-frequency attenders (31.5%; 95% CI 30.7% to 32.3%) than among single attenders (19.4%; 95% CI 19.0% to 19.8%).

**Table 2:** Reasons for ED attendances one time, high frequency and other attenders

Reason for attendance	Number and proportion of attendances made by					
	One-time attenders (N=33 846)	%	High frequency attender in both years (N=1369)	%	Other attenders (N=35 928)	%
Injury	16 019	47.3	4192	34.9	45 973	44
Respiratory conditions	1364	4	1263	10.5	6951	6.7
Infection/infectious disease	1178	3.5	520	4.3	4341	4.2
Gastrointestinal conditions	911	2.7	527	4.4	3487	3.3
Ear, nose and throat conditions	968	2.9	347	2.9	3542	3.4
Central nervous system conditions	351	1	450	3.7	1699	1.6
Urinary conditions	175	0.5	61	0.5	495	0.5
Cardiac/vascular conditions	205	0.6	56	0.4	564	0.5
Mental health	29	0.09	35	0.3	130	0.1
Diabetes/endocrine	12	0.04	16	0.1	30	0.03
Other conditions	1359	4	500	4.2	4229	4.2
Missing	11 269	33.3	4039	33.6	32 998	32
Total	33 846	100	12 016	100	104 469	100

ED, emergency department.

### Heterogeneity of reasons for ED attendance

Of the 71 143 individuals, 18 098 (25.4%) were excluded from the Herfindahl index analysis due to missing diagnosis codes. The final analysis included 1199 individuals who made 7+attendances during their 2-year observation period. Figure 2 illustrates the relationship between Herfindahl index values and age/deprivation status. Individuals aged 8–12 had higher Herfindahl index scores, indicating lower heterogeneity in reasons for attendance. In contrast, infants under age 1 had lower index scores, suggesting greater heterogeneity. While age was associated with heterogeneity, no significant associations were found between Herfindahl index and sex or IMD status (see online supplemental appendix).

## Discussion

### Summary of principal findings

This study retrospectively analysed ED attendances made by 71 143 C&YP aged 15 and under over a 2-year period. Although only a minority (13.6%) were classified as high-frequency attenders in the first year, over half of these individuals (55.1%) continued to use ED services in the second year, and 14.1% remained high-frequency attenders across both years. These high-frequency users were more likely to come from the most deprived IMD quintiles. Analysis of attendance reasons revealed age-related differences in heterogeneity. Children aged 8–12 were more likely to attend for injury-related issues and exhibited lower heterogeneity in their reasons for attendance. In contrast, infants under the age of one had more diverse reasons for attending, with a higher proportion of illness-related visits. These findings suggest that age plays a key role in shaping both the frequency and nature of ED use among C&YP.

### Strengths and limitations

A major strength of this study is the use of a large, multiyear dataset (CUREd), which enabled the analysis of longitudinal ED attendance patterns across a diverse population in Yorkshire and Humber. The consistent 2-year follow-up period for each individual allowed for fair comparisons across the cohort. However, the study also faced several limitations. A key challenge was the inconsistency in primary diagnosis coding across EDs and time periods. Although a mapping framework was developed to align ICD-10 and HES codes, this process may have introduced classification bias. Additionally, a substantial proportion of ED attendances lacked primary diagnosis codes, limiting the number of EDs included in the analysis and potentially introducing selection bias. Importantly, some high-frequency attenders had no diagnosis codes recorded across their entire attendance history. This pattern of missing not at random data could bias analyses of diagnostic trends and highlights the need for improved data quality in ED records. In addition to this, a further limitation was the potential selection bias caused due to the exclusion of hospitals with incomplete diagnostic information. Systematic differences between hospitals with high versus low data completeness—such as differences in population served, case mix or clinical coding systems may have influenced the representativeness of the findings. Finally, the data used in this study predate the COVID-19 pandemic and therefore do not reflect more recent shifts in healthcare-seeking behaviour among C&YP.

### Comparison with other studies

Previous research on adult ED users has shown that heterogeneity in reasons for attendance tends to increase with the number of visits.^21^ The study similarly found that heterogeneity increased in those with a greater number of ED attendances (see appendix). However, when adjusting for attendance frequency, heterogeneity was found to be relatively stable across age groups. In contrast, our findings suggest that among C&YP, age is a significant factor in the diversity of ED use. Infants exhibited greater heterogeneity in reasons for attendance, likely reflecting a broader range of acute health concerns, developmental vulnerabilities, greater levels of parental concern and lack of ability for younger children to explain symptoms. Older children, particularly those aged 8–12, were more likely to attend for injuries, suggesting a more specific pattern of ED use. These differences may reflect developmental, behavioural and environmental factors. For example, older children may be more exposed to injury risks through sports or outdoor activities, while infants may present with a wider range of undifferentiated symptoms that prompt ED visits. One systematic review of qualitative research by Huhtakangas *et al* examined frequent attenders’ experiences of interactions with healthcare personnel, offering valuable insights into their lived experiences.^22^ The review found that high-frequency attenders often felt their personal expertise and lived experience of their condition were undervalued in clinical encounters, particularly when compared with the professional expertise of healthcare providers. This lack of recognition hindered collaborative care efforts and contributed to unresolved health concerns, perpetuating cycles of repeated service use. Although the study focused on adult frequent attenders, it raises important considerations for parents and caregivers of C&YP who frequently attend EDs. These caregivers may similarly experience marginalisation in clinical decision-making, suggesting a need for future qualitative research to explore their perspectives and experiences more deeply.

### Implications for practice and further research

This study highlights the need for targeted interventions to address persistent high-frequency ED use among C&YP, particularly those from more deprived backgrounds. Understanding the underlying drivers of repeated ED attendance—whether related to chronic conditions, unmet primary care needs or social determinants—requires further investigation. Qualitative research, including interviews with parents and caregivers, could provide valuable insights into the decision-making processes behind ED use. Additionally, longitudinal studies tracking ED use into adolescence and early adulthood could help identify critical transition points and inform age-appropriate interventions. The observed age-related variation in heterogeneity also has practical implications. C&YP with repeated injury-related attendances may benefit from safeguarding assessments or injury prevention interventions. Developing a screening tool to flag individuals with an unusually high proportion of injury-related visits could support early identification of at-risk children. The Herfindahl index provides a way of summarising the similarity (or dissimilarity) of recent attendances. This could complement the focus on the presenting problem which characterises most ED work. As such, it could be used to test hypotheses linking heterogeneity to important outcomes such as delayed/missed diagnoses or to monitor safeguarding concerns. It could also examine the possibility that high heterogeneity (repeated attendance with high varied complaints) is a marker for significant parental anxiety or limited coping capacity.

## Conclusions

This study demonstrates that a notable proportion of C&YP are frequent ED users over a 2-year period, with persistent use more common among those from deprived backgrounds. It also introduces a method for quantifying heterogeneity in reasons for attendance, revealing important age-related patterns. These findings can inform future research and service planning aimed at improving care for high-frequency ED users among C&YP.

## Supplementary material

10.1136/bmjpo-2025-003907 online supplemental material 1

## Data Availability

Data may be obtained from a third party and are not publicly available.
