# Supplementary material for: High frequency emergency department use and heterogeneity of reasons for attendance by children and young people: a retrospective cohort study
Source: BMJ Paediatr Open. 2026 Feb 26;10(1):e003907. doi: 10.1136/bmjpo-2025-003907 (PMC12958937; doi:10.1136/bmjpo-2025-003907)
Supplement: online supplemental material 1 [file bmjpo-10-1-s001.docx]

Supplementary Material

Appendix A-Mapping reasons for attendance using ICD-10 HES codes

| **Reason for Attendance** | **ICD-10 Codes** | **ICD-10 Category** | **HES Codes** | **HES Category** |
| --- | --- | --- | --- | --- |
| Injury | **S codes**  **T codes**  **M79.9** | Injury, poisoning and certain other consequences of external causes  Soft tissue disorder, unspecified  Injury non-mental health) | **01-16** | Laceration, Contusion/abrasion, Soft tissue inflammation, Head injury, Dislocation/fracture/joint injury/amputation, Sprain/ligament injury, Muscle/tendon injury, Nerve injury, Vascular injury, Burns and scalds, Electric shock, Foreign body, Bites/stings, Poisoning (inc overdose), Near drowning, Visceral injury |
| Respiratory Conditions | **J codes** | Diseases of the respiratory system | **25** | Respiratory conditions |
| Infections and infectious diseases | **A codes**  **B codes**  **L08.9** | Certain infectious and parasitic diseases  Local infection of skin and subcutaneous tissue, unspecified | **17**  **18**  **19** | Infectious disease  Local infection  septicaemia |
| Gastrointestinal Conditions | **K codes**  **R10.0** | Diseases of the digestive system  Abdominal and pelvic pain | **26** | Gastrointestinal conditions |
| ENT Conditions | **H codes** | Diseases of the eye and adnexa  Diseases of the ear and mastoid process | **34** | ENT conditions |
| Central Nervous System conditions | **G codes** | Diseases of the nervous system | **24** | Central nervous system conditions (exc stroke) |
| Urinary Conditions | **N30-N39** | Symptoms and signs involving the urinary system | **27** | Urinary conditions |
| Cardiac and Vascular conditions | **I codes** | Diseases of the circulatory system | **20**  **21**  **22**  **23** | Cerebro-vascular conditions  Other vascular conditions  Haematological conditions |
| Mental Health | **F codes**  **R45**  **X71 – X83** | Mental and behavioural disorders and self harm | **35**  **37** | Psychiatric  Social problems |
| Diabetes/Endocrine conditions | **E codes**  **E10-E14** | Diabetes mellitus | **301**  **302** | Diabetes  Non-diabetic |
| Other conditions | **R50-R69**  **All other codes left after above** | General symptoms and signs | **31**  **32**  **33**  **36** | Dermatological  Allergy (inc anaphylaxis)  Faciomaxillary conditions  Opthalmological |
| Missing | Missing ‘none’ |  | 38 or 39 or missing |  |

Appendix B- Coefficients of Linear Regression for Heterogeneity of Reasons for Attendance

|  | Coefficient | 95% CI |
| --- | --- | --- |
| Sex |  |  |
| Male (REF) | 0 | - |
| Female | -0.02 | (-0.04, 0) |
|  |  |  |
| Age |  |  |
| <1 (REF) | 0 | - |
| 1-4 | 0.02 | (0, 0.05) |
| 5-7 | 0.12 | (0.07, 0.16) |
| 8-12 | 0.22 | (0.19, 0.25) |
| 13-15 | 0.12 | (0.09, 0.15) |
|  |  |  |
| Deprivation |  |  |
| 5 (REF) | 0 | 0 |
| 4 | 0 | (-0.06, 0.05) |
| 3 | 0.04 | (-0.01, 0.09) |
| 2 | 0.01 | (-0.04, 0.05) |
| 1 | -0.01 | (-0.05, 0.03) |
|  |  |  |
| N | 1194 | |

Appendix C-Sensitivity analysis exploring association between herfindahl index and total attendances made-vertical lines indicate the region in which the author defined the Herfindahl to be stable. To the left of the chosen threshold, the number of attendances was insufficient for meaningful interpretation, whereas to the right, the limited number of individuals led to a substantially reduced sample size and imprecise estimates with wide confidence intervals.


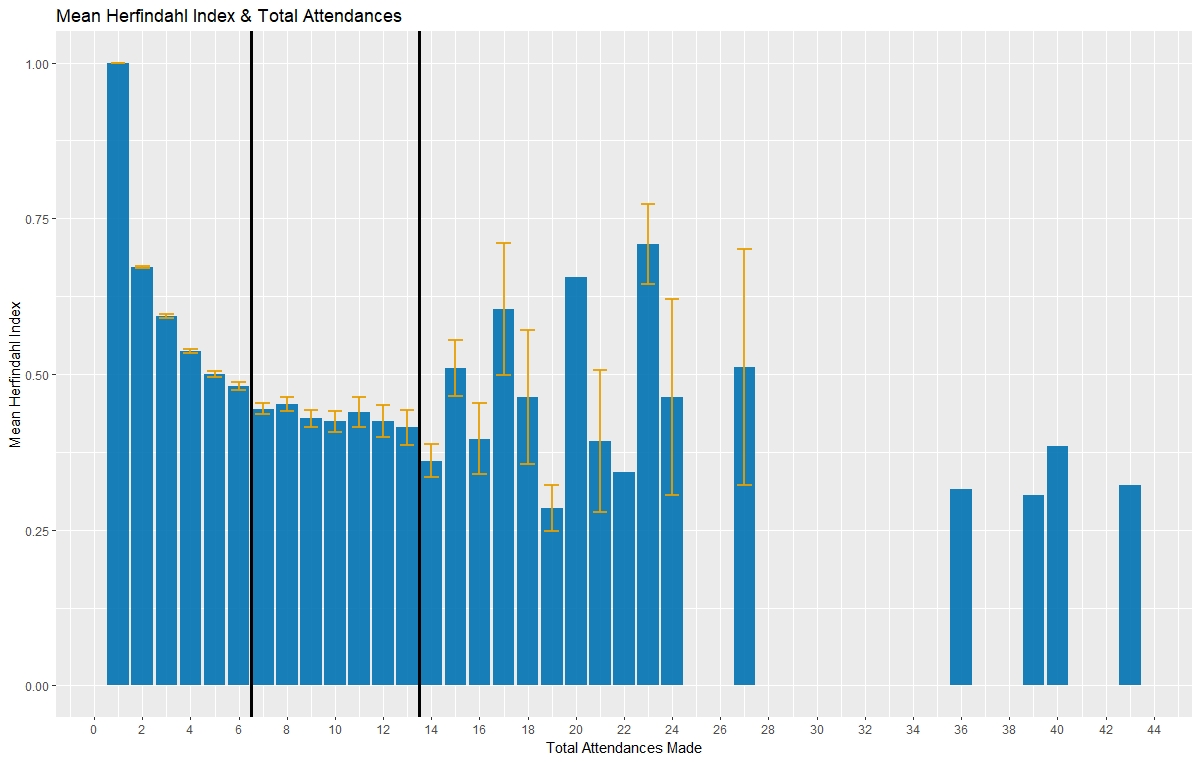

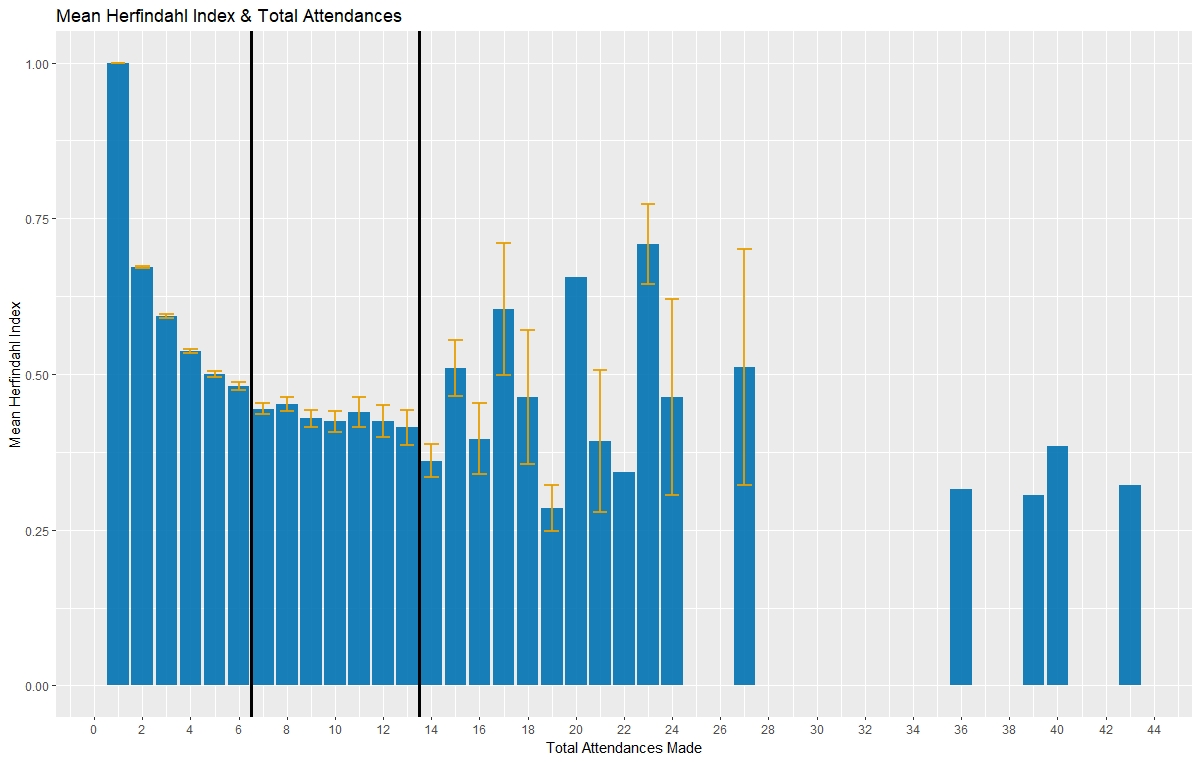


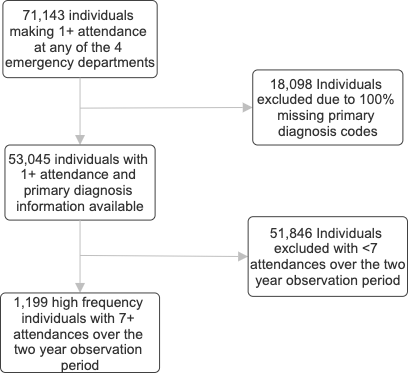


Appendix D- Flowchart of heterogeneity analysis cohort derivation
