# Supplementary material for: High frequency emergency department use and heterogeneity of reasons for attendance by children and young people: a retrospective cohort study
Source: BMJ Paediatr Open. 2026 Feb 26;10(1):e003907. doi: 10.1136/bmjpo-2025-003907 (PMC12958937; doi:10.1136/bmjpo-2025-003907)
Supplement: Supplementary file 2 [file bmjpo-2025-003907.reviewer_comments.pdf]

PEER REVIEW HISTORY

BMJ Paediatrics Open publishes all reviews undertaken for accepted manuscripts. Reviewers are asked to complete a checklist review form and are provided with free text boxes to elaborate on their assessment. These free text comments are reproduced below.

ARTICLE DETAILS

**TITLE (PROVISIONAL)**	High Frequency Emergency Department Use and Heterogeneity of Reasons for Attendance by Children & Young People: A Retrospective Cohort Study
**AUTHORS**	Kumar, Akshay<br>Simpson, Rebecca<br>Husk, Kerryn<br>Johnson, Graham D<br>Burton, Chris

VERSION 1 – REVIEW

**REVIEWER NAME**	Blair, Mitch
**REVIEWER AFFILIATION**	Imperial College London
**REVIEWER CONFLICT OF INTEREST**	No Conflict of Interest
**AI DISCLOSURE**	No, I have not used AI
**DATE REVIEW RETURNED**	02-Sep-2025

**GENERAL COMMENTS**	This paper is a description of frequent attendance by children in ED and its value lies in the fact that it is a large area for analysis and extended over a two year period. In addition it has used a unique method of identifying heterogeneity of reasons for attendance. The authors need to explain to the reader why only 4 of 13 participating hospitals had sufficiently good data for this analysis. In addition, there may be systematic differences between units with or without good data completeness.<br>The APpx c figure needs to have teh vertical lines explained more clearly in teh figure.I would wish to see an expanded discussion on teh utility of this index in other studies. What research questions could be better answered with its use? Also some further expansion in teh discussion around teh already existing contribution made by qualitative literature should strengthen the piece.

**REVIEWER NAME**	Greber-Platzer, Susanne
**REVIEWER AFFILIATION**	Medical University of Vienna, Division of Pediatric Pulmology, Allergology and Endocrinology, Department of Pediatrics and Adolescent Medicine
**REVIEWER CONFLICT OF INTEREST**	No Conflict of Interest
**AI DISCLOSURE**	No, I have not used AI
**DATE REVIEW RETURNED**	01-Oct-2025

**GENERAL COMMENTS**	Dear authors,<br>thanks for the profound work in pediatric ED related to frequent attendance and showing disease related reasons. In literature most

	publications show follow ups for a time period of one year, therefore the main aim of this study was to give an overwiew for a time period of 2 years.<br><br>There are some descriptions on ED related definitions commonly used in UK, but not known in other european countries. Therefore it`s important to explain the categorization IMD, moreover the classification system in type 1 ED, type 2 ED or other types of ED (could also be linked to a reference!).<br><br>Please don`t use abbreviations in the abstract, like IMD, C&YP, because these abbreviations are not known and used in other european countries.<br><br>The main aims are:<br>1.) Measure ED attendance patterns among C&YP over a two-year period.<br>2) Identify common reasons for ED attendances among high-frequency attenders.<br>3) Examine the heterogeneity of reasons for ED use among high-frequency attenders.<br>I think all 3 aspects are worked out in the manuscript, but nevertheless there are decreasing numbers of patients between 1), 2) and 3), because nearby 1/3 (32.5%) of patients had no diagnosis code, which would be most important for 2) and 3). For this it would be helpful to create a flowchart showing patients included and excluded (prepared as Figure!). At least there is only a description in the chapter Results, Patient and Attendance Characteristics, Reasons for ED Attendance and Heterogeneity of Reasons for ED Attendance.<br><br>Is it possible to explain, why not all patients from each hospital had a diagnosis, because it seems that diagnosis should be mandatory, hospital budgets are based on diagnosis and examination coding systmes.<br><br>Chapter Cohort Preparation: It`s necessary to explain, why 7 hospitals are not coding diagnosis, the most important and relevant information on patient illnesses attending EDs. Please write an clear explanation for understanding the UK coding system for pediatric EDs.<br>High-frequency attenders were defined as individuals with ≥3 attendances in a given 12-month period. - only for clarification: ≥3 attendances, for each of both years!<br><br>Please perform consistency in your descriptions, writings concerning the upper age limit are: aged 15 and under / aged ≤15 years / under the age of 16 - it would be more easy to use the same writing for all!<br><br>Chapter Reason for Attendance: Codes of length 1, 2 or 3 were omitted due to<br>ambiguity (as these codes may have represented anatomical side and/or area).<br>Does this mean there are still patients with inadequate diagnosis codes using a national certified system? Please can you explain how this can be valid! On the other hand the exclusion of patients with an inadequate diagnosis are a pitty, because you are missing important data. For understanding the HES classification and the coding of insufficient diagnosis, and not using an international classification systems.

	Please write more details, thanks.<br><br>Chapter Heterogeneity of Reasons for ED Attendance<br>This great bias must be explained, because therefore missing patients couldn`t be diagnosed in the group of injuries or illnesses! This could lead to exclusion of patients having no sufficient diagnosis code by HES maybe only for one ED visit, also if the patient attended several times and had sufficient diagnosis coding!<br><br>In summary the manuscript is well written and shows frequent attendance related to injuries, illnesses, deprivated areas, age and gender, which seems to be very informative and maybe will help to establish a graduated healthcare system also for acute illnesses and minor injuries in childhood and adolescents.<br><br>Kind regards!

VERSION 1 – AUTHOR RESPONSE

Reviewer: 1

- **This paper is a description of frequent attendance by children in ED and its value lies in the fact that it is a large area for analysis and extended over a two year period. In addition it has used a unique method of identifying heterogeneity of reasons for attendance.**

Thank you for these positive comments. We appreciate the reviewer’s recognition of the study’s scale, the two-year observation period, and the novel method used to identify heterogeneity in reasons for attendance.

- **The authors need to explain to the reader why only 4 of 13 participating hospitals had sufficiently good data for this analysis.**

Thank you for this comment. Nine of the thirteen hospitals included in our study had insufficient primary diagnosis coding for patients. This was due to several factors: suboptimal diagnosis code entry by clinicians, changes in diagnosis coding systems at some hospitals during the study period, and limited information provided under the older HES coding framework. We agree that consistent use of standardised diagnosis coding and accurate data entry would enhance the utility and robustness of the method described in the manuscript.
“This was due to several factors: suboptimal diagnosis code entry by clinicians, changes in diagnosis coding systems at some hospitals during the study period, and limited information provided under the older hospital episode statistics (HES) coding framework.”

- **In addition, there may be systematic differences between units with or without good data completeness.**

Thank you for this comment. We acknowledge that systematic differences may exist between hospitals with higher versus lower data completeness, which could introduce bias into our analyses. Unfortunately, due to the limitations of the available dataset, we were unable to fully assess these differences. We have now added this as a limitation in the Discussion.

“In addition to this, a further limitation was the potential selection bias caused due to the exclusion of hospitals with incomplete diagnostic information. Systematic differences between hospitals with high versus low data completeness—such as differences in population served, case mix, or clinical coding systems—may have influenced the representativeness of the findings.”

- **The APpx c figure needs to have the vertical lines explained more clearly in the figure.**

Thanks for pointing this out. The lines indicate the region in which the Herfindahl index was utilised in relation to total attendances made. As this study is interested in exploring high frequency attendance over the two year period we considered a lower threshold of >6 and an upper threshold of <14 due to

minimal sample size of individuals with 15+ attendances over the two years. This information has now been included in the caption of the figure.
"To the left of the chosen threshold, the number of attendances was insufficient for meaningful interpretation, whereas to the right, the limited number of individuals led to a substantially reduced sample size and imprecise estimates with wide confidence intervals."

- **I would wish to see an expanded discussion on the utility of this index in other studies. What research questions could be better answered with its use?**

Thank you for this suggestion. We have now added some detail to the discussion outlining how the index can be utilised in future studies.
"The Herfindahl index provides a way of summarizing the similarity (or dissimilarity) of recent attendances. This could complement the focus on the presenting problem which characterises most ED work. As such, it could be used to test hypotheses linking heterogeneity to important outcomes such as delayed/missed diagnoses or to monitor safeguarding concerns. It could also examine the possibility that high heterogeneity (repeated attendance with high varied complaints) is a marker for significant parental anxiety or limited coping capacity. "

- **Also some further expansion in the discussion around the already existing contribution made by qualitative literature should strengthen the piece.**

Although there is limited qualitative research conducted exploring the experiences of C&YP high frequency users (or their parents/caregivers) we have now added a qualitative study exploring the lived experiences of adult high frequency attenders and linked this to parents and caregivers.
"One systematic review of qualitative research by Huhtakangas et al. (2021) examined frequent attenders' experiences of interactions with healthcare personnel, offering valuable insights into their lived experiences. The review found that high-frequency attenders often felt their personal expertise and lived experience of their condition were undervalued in clinical encounters, particularly when compared to the professional expertise of healthcare providers. This lack of recognition hindered collaborative care efforts and contributed to unresolved health concerns, perpetuating cycles of repeated service use. Although the study focused on adult frequent attenders, it raises important considerations for parents and caregivers of C&YP who frequently attend emergency departments. These caregivers may similarly experience marginalisation in clinical decision-making, suggesting a need for future qualitative research to explore their perspectives and experiences more deeply."

Reviewer: 2

- **Dear authors, thanks for the profound work in pediatric ED related to frequent attendance and showing disease related reasons. In literature most publications show follow ups for a time period of one year, therefore the main aim of this study was to give an overview for a time period of 2 years.**

Thank you for your kind comments. We appreciate the reviewer's recognition of our work and agree that extending the follow-up period to two years provides a broader perspective on patterns of frequent attendance and associated attendance reasons in emergency departments.

- **There are some descriptions on ED related definitions commonly used in UK, but not known in other european countries. Therefore its important to explain the categorization IMD, moreover the classification system in type 1 ED, type 2 ED or other types of ED (could also be linked to a reference!).**

Thank you for this comment we have now relevant references about the UK specific definitions mentioned in the text.
Reference list

1) ***Abel GA, Barclay ME and Payne RA. Adjusted indices of multiple deprivation to enable comparisons within and between constituent countries of the UK including an illustration using mortality rates. BMJ open 2016; 6: e012750.***
2) ***https://www.england.nhs.uk/statistics/wp-content/uploads/sites/2/2019/07/AE-Attendances-Emergency-Definitions-v4.0-final-July-2019.pdf***

- **Please dont use abbreviations in the abstract, like IMD, C&YP, because these abbreviations are not known and used in other european countries.**

We have now omitted the UK specifc abbreviations in the abstract, however we have kept ED after defining it. See abstract.

- The main aims are:
  1.) Measure ED attendance patterns among C&YP over a two-year period.
  2) Identify common reasons for ED attendances among high-frequency attenders.
  3) Examine the heterogeneity of reasons for ED use among high-frequency attenders.
  I think all 3 aspects are worked out in the manuscript, but nevertheless there are decreasing numbers of patients between 1), 2) and 3), because nearby 1/3 (32.5%) of patients had no diagnosis code, which would be most important for 2) and 3). For this it would be helpful to create a flowchart showing patients included and excluded (prepared as Figure!).

Thank you for this suggestion, we have now included a flow chart of the number of patients included/excluded in the appendix.

- **Is it possible to explain, why not all patients from each hospital had a diagnosis, because it seems that diagnosis should be mandatory, hospital budgets are based on diagnosis and examination coding systems. Chapter Cohort Preparation: It`s necessary to explain, why 7 hospitals are not coding diagnosis, the most important and relevant information on patient illnesses attending EDs.**

Nine of the thirteen hospitals included in our study had insufficient primary diagnosis coding for patients. This was due to several factors: suboptimal diagnosis code entry by clinicians, changes in diagnosis coding systems at some hospitals during the study period, and limited information provided under the older HES coding framework. We agree that consistent use of standardised diagnosis coding and accurate data entry would enhance the utility and robustness of the method described in the manuscript. We have now added this explanation to section x.

"Due to low rates of primary diagnosis coding in some EDs, only EDs with ≥70% non-missing primary diagnosis codes were included. This was due to several factors: suboptimal diagnosis code entry by clinicians, changes in diagnosis coding systems at some hospitals during the study period, and limited information provided under the older Hospital Episode Statistics (HES) coding framework. This resulted in four EDs (two university teaching hospitals and two district general hospitals) being retained."

- **Please write an clear explanation for understanding the UK coding system for pediatric EDs.**

Thank you this comment. The authors have now addressed this and outline the different primary diagnosis coding systems employed by English EDs over the period of interest.

"Between 2014 and 2017, three diagnosis coding systems were employed across EDs in England: the legacy HES A&E 5- or 6-character codes used for ED-specific reporting; ICD-10 (International Classification of Diseases) codes, primarily applied to admitted patients; and Systematised Nomenclature of Medicine – Clinical Term (SNOMED CT), introduced with the Emergency Care Data Set (ECDS) in 2017 but not yet routinely adopted during this period."

- **High-frequency attenders were defined as individuals with ≥3 attendances in a given 12-month period. - only for clarification: ≥3 attendances, for each of both years!**

Thank you for this comment, as you mention for the heterogeneity analysis we only included individuals who were frequent attenders in both observation years. However we also display in the figures those who are high frequency in either of the two observation years.

- **Please perform consistency in your descriptions, writings concerning the upper age limit are: aged 15 and under / aged ≤15 years / under the age of 16 - it would be more easy to use the same writing for all!**

Thank you for this comment we have now consistently used the term "aged 15 and under" to avoid confusion.

- **Chapter Reason for Attendance: Codes of length 1, 2 or 3 were omitted due to ambiguity (as these codes may have represented anatomical side and/or area). Does this mean there are still patients with inadequate diagnosis codes using a national certified system? Please can you explain how this can be valid! On the other hand the exclusion of patients with an inadequate diagnosis are a pitty,**

because you are missing important data. For understanding the HES classification and the coding of insufficient diagnosis, and not using an international classification systems.
Please write more details, thanks.

Thank you for this comment, the legacy hes coding system was designed to be 5/6 digits, the first 2/3 digits referring to the primary diagnosis, the next 2 digits referring to the anatomical site and the final digit relating to the anatomical side. Unfortunately it was often the case that clinicians input two digit codes which could correspond to the condition or anatomical site which caused the ambiguity and hence were ommitted.

Chapter Heterogeneity of Reasons for ED Attendance

- **This great bias must be explained, because therefore missing patients couldn't be diagnosed in the group of injuries or illnesses! This could lead to exclusion of patients having no sufficient diagnosis code by HES maybe only for one ED visit, also if the patient attended several times and had sufficient diagnosis coding!**

This bias is outlined in the limitations and outlines how future research could apply this method to data with more homogeneous diagnosis coding systems.
"Additionally, a substantial proportion of ED attendances lacked primary diagnosis codes, limiting the number of EDs included in the analysis and potentially introducing selection bias."

- **In summary the manuscript is well written and shows frequent attendance related to injuries, illnesses, deprivated areas, age and gender, which seems to be very informative and maybe will help to establish a graduated healthcare system also for acute illnesses and minor injuries in childhood and adolescents.**

Thank you for the kind words and taking the time to review the manuscript.

VERSION 2 – REVIEW

**REVIEWER NAME**	Greber-Platzer, Susanne
**REVIEWER AFFILIATION**	Medical University of Vienna, Division of Pediatric Pulmology, Allergology and Endocrinology, Department of Pediatrics and Adolescent Medicine
**REVIEWER CONFLICT OF INTEREST**	No Conflict of Interest
**AI DISCLOSURE**	No, I have not used AI
**DATE REVIEW RETURNED**	29-Jan-2026

**GENERAL COMMENTS**	Thanks very much for your improvements and explanations clarifying the problems of the different coding systems and therefore the missing diagnosis and limitations of your work.
